# Comparisons of air-conduction hearing thresholds between manual and automated methods in a commercial audiometer

**DOI:** 10.3389/fnins.2023.1292395

**Published:** 2023-12-21

**Authors:** Hui Liu, Xinxing Fu, Mohan Li, Shuo Wang

**Affiliations:** ^1^Department of Otolaryngology, Head and Neck Surgery, Beijing Tongren Hospital, Capital Medical University, Beijing, China; ^2^Key Laboratory of Otolaryngology, Head and Neck Surgery, Ministry of Education, Beijing Institute of Otolaryngology, Beijing, China; ^3^Medical School, The University of Western Australia, Crawley, WA, Australia; ^4^Ear Science Institute Australia, Subiaco, WA, Australia

**Keywords:** automated audiometry, pure-tone audiometry, KUDUwave, response time, non-soundproof booth

## Abstract

**Objective:**

To investigate the correlation of air-conduction thresholds between automated audiometry in a non-isolated environment and manual audiometry in participants with normal hearing and different degrees of hearing loss.

**Methods:**

Eighty-three participants aged 11–88 years old underwent automated pure-tone audiometry in a non-acoustically isolated environment, and the results were compared with those of manual pure-tone audiometry performed in a standard acoustically isolated booth, with the order of testing randomised. Six frequencies of 250, 500, 1,000, 2000, 4,000 and 8,000 Hz were tested.

**Results:**

All 166 ears were completed and 996 valid hearing threshold data were obtained, with 28 data exceeding the 95% confidence interval in the Bland–Altman plot, accounting for 2.81% of all data. The means and standard deviations of the differences for the six frequencies from 250 to 8,000 Hz were, respectively, 0.63 ± 5.31, 0.69 ± 4.50, 0.45 ± 4.99, 0.3 ± 6.2, −0.15 ± 4.8, and 0.21 ± 4.97 dB. The correlation coefficients of the two test results for normal hearing, mild, moderate, severe and above hearing loss groups were 0.95, 0.92, 0.97, and 0.96, respectively. The correlation coefficient of the automated and manual audiometry thresholds for the age groups under 40 years, 40–60 years, and 60 years above, were 0.98, 0.97 and 0.97, respectively, with all being statistically significant (*p* < 0.01). The response time of the three age groups were 791 ± 181 ms, 900 ± 190 ms and 1,063 ± 332 ms, respectively, and there was a significant difference between the groups under 40 years and over 60 years.

**Conclusion:**

There was good consistency between automated pure-tone audiometry in a non-acoustically isolated environment and manual pure-tone audiometry in participants with different hearing levels and different age groups.

## 1 Introduction

WHO estimates by 2050, nearly 2.5 billion people will suffer from some degree of hearing loss, of whom at least 700 million will need rehabilitation services (World Health Organization, 2021). In addition to its impact on interpersonal communication, psychosocial well-being and quality of life, hearing loss has a significant socio-economic impact. In children, hearing loss can limit language development and lead to difficulties in social integration and access to education, with significant impacts on the family; in adults, hearing loss can lead to higher unemployment rates and social isolation (Kramer et al., 2006). In older adults, hearing loss is also associated with cognitive decline and dementia (Livingston et al., 2017). In China, according to the results of the second national sample survey of people with disabilities, there are 27.8 million people with hearing disabilities, ranking first among the five major disabilities (Sun et al., 2008). In recent years, the number of people with hearing loss has increased with the increase in population aging. Early detection, diagnosis, and intervention can reduce the socioeconomic burden of hearing loss.

Pure-tone audiometry is the most basic and important method of assessing hearing loss. Traditional manual testing methods for pure-tone audiometry require three conditions to be met: a compliant acoustic isolation room, calibrated audiometers, and professionally trained audiologists. In China, most tertiary hospitals in first and second-tier cities can fulfil these conditions for testing, but in remote and economically underdeveloped areas, there are a limited number of hospitals that can fulfil the conditions for testing, which means that it is difficult for many people to access hearing healthcare, and at the same time, the large group of patients puts tertiary hospitals under even greater pressure.

Automated pure-tone audiometry means hearing threshold testing where the testing process is automated with no or minimal staff involvement (Wasmann et al., 2022). A growing body of research suggests that automated pure-tone audiometry can be useful in mass hearing screening, in remote and economically underdeveloped areas (Visagie et al., 2015; Eksteen et al., 2019; Sandström et al., 2020). There are three approaches to automate audiometry, including software solutions such as the AMTAS (Automated Method for Testing Auditory Sensitivity) and the Home Hearing Test (HHT); hardware solutions such as the KUDUwave portable audiometer; and smartphone/tablet solutions such as the hearScreen and hearTest application (Shojaeemend and Ayatollahi, 2018).

An automated pure tone audiometer for complete diagnostic testing purposes needs to include air conduction testing, bone conduction testing, masking techniques, and controling the noise attenuation. The KUDUwave 5,000 audiometer (hereinafter referred to as KUDUwave) is a portable audiometer that performs air-conducted and bone-conducted pure tone hearing threshold tests in automated and manual modes, with masking when required, by insert earphones covered by circumaural earcups to increase ambient noise attenuation, and continuous monitoring of ambient noise and determination of the amount of attenuation by using microphones inside and outside the circumaural earcups. This combination of attenuation and monitoring allows hearing tests to be performed in non-acoustically isolated environments, ensuring that pure tone thresholds can be tested down to 0 dB HL at maximum permissible ambient noise levels (MPANLs) of 70, 69, 58, 53, 50, 59, and 59 dB SPL for octaves from 0.125-8 k Hz. The audiometers are connected to a computer via a USB port with Internet access for remote hearing tests.

Existing studies reported good correlations between the results of automated and traditional manual pure-tone audiometry, both in adults and children using KUDUwave in sound-insulated and non-insulated environments. Swanepoel et al. conducted automated pure-tone audiometry in non-sound-insulated environments using KUDUwave in 23 adults with normal hearing (Swanepoel De et al., 2015) and in 149 children (Swanepoel De et al., 2013), and obtained reliable results when compared to traditional manual pure-tone audiometry. Maclennan-Smith et al. (Maclennan-Smith et al., 2013) performed automated versus manual testing of the KUDUwave on 147 older adults with normal hearing or varying degrees of hearing loss. The automated test was performed in a normal room, and the manual test was performed in an acoustically insulated room, with 95% of the threshold difference in air-conducted (250–8,000 Hz) and 86% of the threshold difference in bone-conducted (250–4,000 Hz) were within 5 dB. Swanepoel et al. (Swanepoel De et al., 2010a) and Visagie et al. (Visagie et al., 2015) also reported remote pure-tone audiometry using KUDUwave that reliable test results were obtained.

Governder and Mars (Govender and Mars, 2018a) conducted hearing screening in a group of children aged 6–12 years in a rural primary school and those who failed the screening underwent diagnostic audiometry, both screening and diagnostic audiometry were conducted using KUDUwave. The results showed high specificity (100%) but low sensitivity (65.2%) for automated pure-tone audiometric screening. The 1,500 ms suggested by KUDUwave was used as the reaction time, and Governder and Mars concluded that this reaction time might be insufficient for child subjects. It is proposed that the response time of the subjects should be investigated, and the parameters of the device should be adjusted. Storey et al. (Storey et al., 2014) measured 31 subjects (aged 15 to 80 years) with different degrees of hearing loss using the KUDUwave in quiet and noisy environments, most of the thresholds obtained were within ±5 dB of the results of the manual pure-tone audiometry in an acoustic chamber (89 and 92% in quiet and noisy environments, respectively). However, thresholds obtained with the KUDUwave in 5% of the test ears showed large differences compared to clinical audiometers, with differences in thresholds up to 60 dB.

This study analysed subjects of different ages and degrees of hearing loss in groups and reported the response times of subjects of different ages. It is expected to provide evidence for setting parameters for automated pure-tone audiometry.

## 2 Materials and methods

### 2.1 Participants

Eighty-three participants, 41 males and 42 females, aged 11–88 years (median age was 57 years), were enrolled from the clinical audiology centre, Department of Otorhinolaryngology, Head and Neck Surgery, Beijing Tongren Hospital. Inclusion criteria: ability to understand the test requirements and cooperate in completing the test; including normal hearing and varying degrees of sensorineural, conductive and mixed hearing loss. Exclusion criteria: known cognitive impairment and inability to understand the test requirements. This research project was approved by the Medical Ethics Committee for Clinical Research of Beijing Tongren Hospital, and informed consent was obtained from the participants before the tests.

### 2.2 Equipment

Both manual and automated pure-tone audiometry were performed using the KUDUwave 5,000 (GeoAxon, Pretoria) clinical audiometer. KUDUwave was connected to a computer via a USB port, and the test procedure was operated by software installed on a laptop computer. Before testing with the KUDUwave, it was calibrated according to ISO 389-2: 1994. The B&K 2240 (Brüel & Kjær, Denmark) sound level meter was used to monitor the clinic’s environmental noise, recording the average and maximum noise values.

### 2.3 Test methods

An otoscopic examination of the subject’s external ear canal was conducted to remove possible cerumen obstruction. All participants were tested for pure-tone air-conduction hearing thresholds by manual and automated methods, in a randomised order, with adequate rest given between each test. Test frequencies were 250, 500, 1,000, 2000, 4,000 and 8,000 Hz. The test requirements were fully explained to the participant before the tests. The KUDUwave insert earphones were fully into the ear canal and the end flush with the tragus, and then the circumaural earphones were placed over the insert earphones. Before the test, a pure-tone signal sound was given manually for the subject to practice. The subjects were instructed to press the button as soon as they heard the pure tone, and to perform manual or automated audiometry after the subjects had fully understood the test requirements.

Manual pure-tone audiometry was performed in a standard double-walled soundproof booth with the KUDUwave. The manual test determined the hearing threshold according to the method specified in ISO 8253-1:2010. The automated pure-tone audiometry was conducted using the shortened ascending method (ISO 8253-1:2010). The initial sound intensity for each frequency was 30 dB HL, and the sound duration was 1,000 ms. The waiting response time is 2000 ms, i.e., it’s considered to be a valid response to press the transponder button within 2000 ms from the time the tone is given, otherwise it will be marked as a false positive response. At the end of the test, a pure-tone audiogram was automatically generated, while KUDUwave reported the percentage of false positives, the noise monitoring value, the number of times the subject responded to the signal, and the response time to press the button. The automated test was conducted in a general clinic room, and the average and maximum values of ambient noise were monitored with a sound level meter during the test. A comparison of manual and automated hearing testing protocols is shown in eTable 1. To avoid the audiologist referring to the results of the first test for a second test, separate audiologists operated manual and automated tests and were unaware of each other’s results. Meanwhile, in order to minimize the variability, the instructions remained the same between the two audiologists. The test procedure is shown in eFigure 1.

### 2.4 Data processing

Descriptive measures illustrated the difference between the thresholds of manual and automated.

audiometry, described as mean ± SD. An independent samples t-test was performed on the difference in thresholds from 250 to 8,000 Hz obtained by the two testing methods, with *p* < 0.05 as the criterion for significance. Pearson correlation tests were used to assess whether there was a correlation between manual and automated test results. One-way ANOVA was used to compare the thresholds between different age groups and groups with different hearing loss degrees. The post-hoc power analysis was run to confirm the sample size. The difference between the two test methods was analysed using Bland–Altman plots. All statistical analyses were performed by SPSS 25 (SPSS Inc., Chicago, Illinois, USA).

## 3 Result

**Figure 1:**
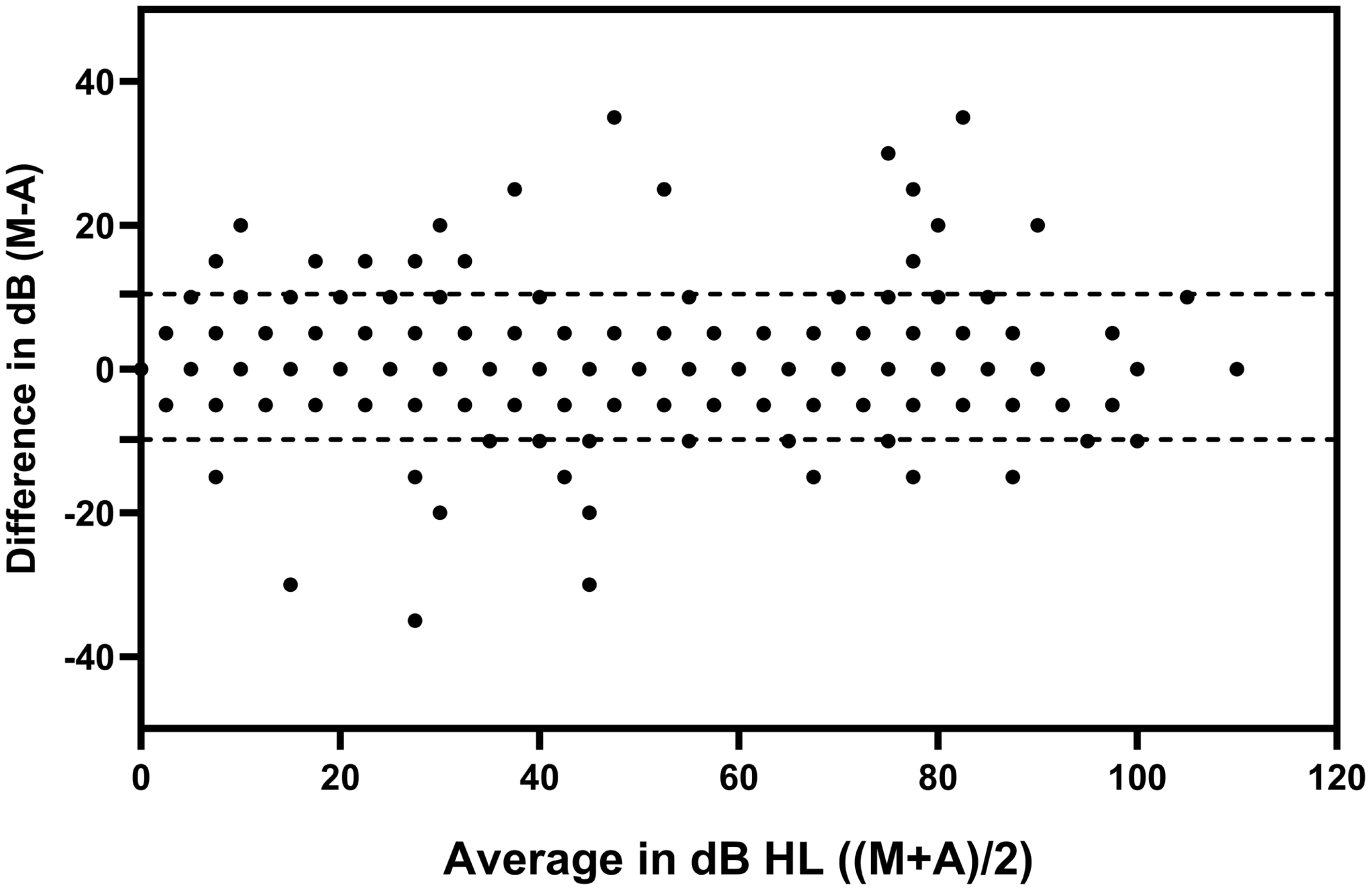
Bland–Altman plots of the results of the automated and manual pure-tone audiometry. The dotted lines in the plots are the upper and lower limits of the 95% confidence intervals. M: manual pure-tone audiometry threshold; A: automated pure-tone audiometry threshold. Dots were pooled across both test ears and all test frequencies.

A total of 166 ears were obtained from 83 participants, with 6 frequencies tested in each ear for a total of 996 data. A total of 28 data exceeded the 95% upper and lower limits of the Bland–Altman plots, accounting for 2.8% of all the data, less than 5%, indicating good consistency between manual and automated pure-tone audiometry results (Figure 1). The Bland–Altman plots of the separate frequencies from 250, 500, 1,000, 2000, 4,000 and 8,000 Hz were listed as supplementary material (eFigures 2A-F). The post-hoc power analysis was run to confirm the sufficient sample size, the calculation results were listed as supplementary material (eTable 2).

The difference between the automated and manual pure-tone audiometry results and the absolute value of the difference were shown in Table 1, the maximum value of the absolute value of the difference at each frequency is between 20 and 35 dB. The distribution of the absolute difference between the manual and the automated thresholds was shown in eFigure 3.

**Table 1:** The difference between manual and automated audiometry thresholds for each frequency.

Hz	250	500	1,000	2000	4,000	8,000	Total
M difference in dB (SD)	0.63 (5.31)	0.69 (4.50)	0.45 (4.98)	0.30 (6.22)	−0.15 (4.84)	0.21 (4.97)	0.36 (5.16)
Abs M difference in dB (SD)	3.16 (4.31)	2.74 (3.63)	2.74 (4.18)	3.31 (5.27)	2.20 (4.32)	2.44 (4.33)	2.77 (4.37)
Maximum of Abs difference	25	20	35	30	35	35	35

M difference: the average value of the difference between the manual and the automated thresholds (manual minus automated values); M difference: the average of the absolute value of the difference between the manual and the automated threshold; Maximum of Abs difference: the maximum of the absolute value of the difference between the manual and the automated threshold.

All participants were divided into four groups according to better ear average hearing level (mean values of hearing thresholds at four frequencies, 500, 1,000, 2000, and 4,000 Hz), the normal group (≤25 dB HL), the mild hearing loss group (26–40 dB HL), the moderate hearing loss group (41–60 dB HL), and the severe and above hearing loss group (≥61 dB HL) (Olusanya et al., 2019). The correlation coefficient (r) between the automated and the manual test in the mild hearing loss group was 0.92 while the correlation coefficients were equal to or greater than 0.95 in all other groups, all with significance (*p* < 0.01). Threshold differences were not statistically significant between the groups (*p* > 0.05). The correlation between the automated and manual test results for participants with different hearing levels is shown in Table 2.

**Table 2:** Correlation of manual and automated pure-tone audiometry thresholds for different hearing levels.

	Groups by hearing level				Total (*n* = 83)
	Normal (*n* = 26)	Mild (*n* = 14)	Moderate (*n* = 30)	Severe and above (*n* = 13)	
M difference in dB (SD)	0.77 (3.94)	1.19 (6.43)	−0.35 (3.60)	−0.76 (5.29)	0.20 (4.60)
r	0.95	0.92	0.97	0.96	0.98
*p*-values	<0.01	<0.01	<0.01	<0.01	<0.01

M difference: the average value of the difference between the manual and the automated thresholds (manual minus automated values); r: correlation coefficient.

All participants were also divided into three groups according to age, the group under 40 years, 40–60 years and over 60 years, and the correlation coefficient (r) between the automated and the manual test for each group was greater than 0.9, all with significance (p < 0.01) (Table 3). The demographic of the participants is shown in eTable 3.

**Table 3:** Correlation between manual and automated pure-tone audiometry thresholds in different age groups.

	Age groups			Total (*n* = 83)
	<40 years (*n* = 21)	40 ~ 60 years (*n* = 27)	>60 years (*n* = 35)	
M difference in dB (SD)	0.83 (4.88)	−0.20 (5.61)	0.50 (4.93)	0.36 (5.16)
r	0.98	0.97	0.97	0.98
*p*-values	<0.01	<0.01	<0.01	<0.01

M difference: the average value of the difference between the manual and the automated thresholds (manual minus automated values); r: correlation coefficient.

The test durations for automated and manual pure tone audiometry were 320 ± 42 s and 281 ± 90 s, respectively, with the manual test time being less than the automatic test time (*p* < 0.05). The average value of ambient noise in the general clinic was 41.5 ± 4.6 dB(A), and the maximum value of ambient noise was 66.2 ± 7.2 dB(A).

**Figure 2:**
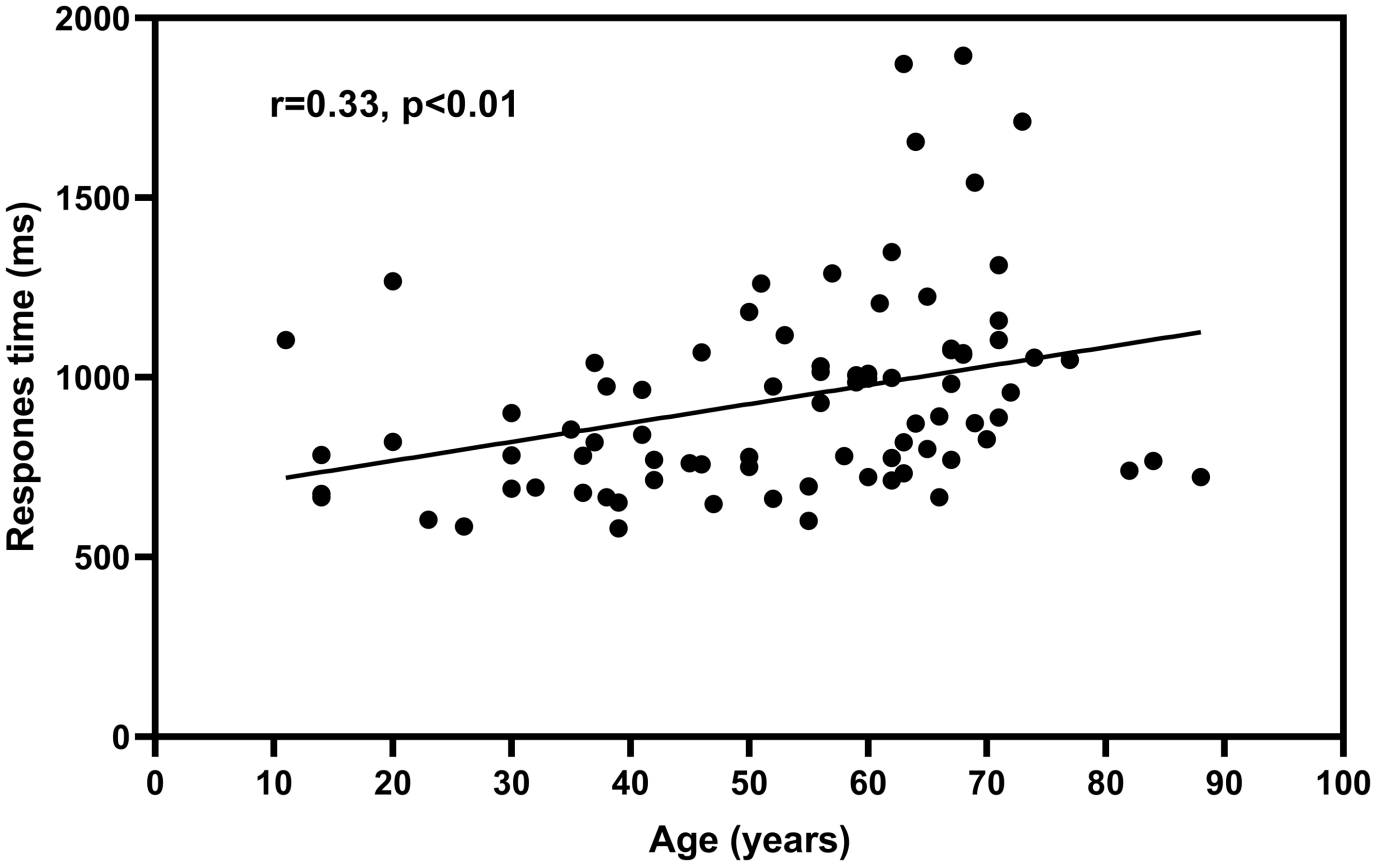
Relationship between participants’ age and response time. The fitted curve in the figure is based on linear regression. Data were pooled across both test ears and all test frequencies.

False positives of automated audiometry were reported from KUDUwave, and the results were listed as supplementary material (eTable 4). The response time for subjects to press the transponder button increased with age (Figure 2), and the overall response time for all subjects was 941.5 ± 279.3 ms. The response time was 791.5 ± 181.2 ms in the age group below 40 years, 900.4 ± 190.9 ms in the age group 40–60 years, and 1063.1 ± 332.3 ms in the group 60 years above. There was a statistical difference in response time between the under 40 years group and the over 60 years group (*p* < 0.01). The results were listed as supplementary material (eTable 5).

## 4 Discussion

In our previous study, a comparison of automated and manual pure-tone audiometry was performed on normal hearing subjects, and a good correlation was found between the results of the two tests (Liu et al., 2021), which is consistent with the findings of other studies (Swanepoel De et al., 2010b; Mahomed et al., 2013; Corry et al., 2017). However, there are fewer studies on the correlation between automated and manual pure-tone audiometry in participants with hearing loss. Measurement bias might be introduced by including only participants with normal hearing (Rutjes et al., 2006). Participants with normal hearing are known to have hearing within a specific range, which will reduce the possible range of variation between the two diagnostic techniques. Reducing the measurement bias by testing on clinical patients would provide a more valid estimate of the accuracy of automated pure-tone audiometry in practice (Whitton et al., 2016). The participants in this study were drawn from the clinical audiology centre, covering a wide range of hearing loss conditions of varying degrees and natures, and the range of ages is large, simulating the more common situations that occur in clinical audiometry.

The overall consistency between automated and manual audiometry was good, with an outlier of 2.8%, less than 5%. The absolute maximum value of the threshold difference between the two methods ranged from 20 to 35 dB. These results are consistent with previous studies that have shown strong benefits of automated pure-tone audiometry in screening for large-scale hearing modalities (Mahomed et al., 2013; Wasmann et al., 2022).

In this study, the correlation between automated and manual pure-tone audiometry was comparable in the groups with different hearing levels, demonstrating that automated audiometry can obtain reliable results in people with various degrees of hearing loss. In other studies of automated pure-tone audiometry, due to the difficulty of controlling ambient noise and the calibration of headphones, the application scenario is mainly for self-hearing healthcare monitoring at home, which is not available for screening mild hearing loss (Whitton et al., 2016; Sandström et al., 2020; Wasmann et al., 2022). There are a limited number of studies on automated pure-tone audiometry in participants with moderate and above hearing loss, where subjects are not grouped by degree of hearing loss. Brennan-Jones recruited 42 participants with different degrees of hearing loss to conduct a correlation study between automated and manual pure tone audiometry, with 86.5% of the thresholds differing within 10 dB, and 94.8% of the thresholds differing within 15 dB, which is similar to this study, but no subgroup analysis of the degree of hearing loss was performed (Brennan-Jones et al., 2016). Whitton (Whitton et al., 2016) performed automated pure tone audiometry on 19 subjects with varying degrees of hearing loss, finding higher thresholds at 250 Hz when collected at home, and attributing this to background noise in the home environment, but did not group the degrees of hearing loss to see if this phenomenon occurred only in subjects with specific levels of hearing loss. Tonder, Govender, and Bornman all performed automated pure-tone audiometry of participants with different degrees of hearing loss, but none of them performed detailed subgroup analyses based on the degree of hearing loss (Van Tonder et al., 2017; Bornman et al., 2018; Govender and Mars, 2018b).

In the three age groups, the thresholds correlated well between automated and manual tests, with correlation coefficients above 0.9, confirming that automated audiometry can be carried out in the elderly population. Margolis et al. performed automated pure-tone audiometry in a non-isolated environment on 28 older adults with a mean age of 65 years, and the hearing thresholds were slightly higher than those of manual pure-tone audiometry obtained in a sound-isolation room, but no statistical differences were observed (Margolis et al., 2016). In a similar study by Mosley, the mean hearing thresholds for four frequencies were correlated between automated and manual pure-tone audiometry in 112 older adults aged 60 years or older, as well as in different degrees of hearing loss (Mosley et al., 2019).

In addition to false-positive responses, which are the most common phenomenon affecting the reliability of test results, observing other indicators can help determine the test reliability. Margolis (Margolis et al., 2007) suggested a method for predicting the accuracy of automated audiometry thresholds (Qualind™), a multiple regression analysis of eight factors associated with test accuracy, including masked alarm rate, time per trial, false-positive rate, false-negative rate, mean test–retest variance, the number of air-bone gaps >50 dB, the number of air-bone gaps <−10 dB, and the mean air-bone gap, yielded a regression coefficient of 0.84. Not all of these eight factors were available in this study and therefore could not be cross-validated with Margolis’ results. Therefore, more metrics with higher sensitivity and specificity still need to be explored for validation of individual quality control in automated pure-tone audiometry.

The response time of the participants to press the transponder button after hearing the sound was positively correlated with age, and the overall response time was 941.5 ± 279.3 ms. Significant differences were observed between the groups under 40 years and over 60 years, which may be explained by the gradual decline of brain function with age. The reaction time for all subjects in this study was set to 2000 ms or less; if the reaction time is set too long, a portion of the false positives may be included in the correct response, which will affect the ability to obtain accurate automated audiometric results. Samantha et al. (Govender and Mars, 2018a) set the reaction time to 1,500 ms in a group of children aged 6–12 years old for hearing screening, and the authors concluded that the reaction time may be insufficient for child subjects. The results of this study showed that the reaction time did not exceed 1,500 ms for all subjects. Still, the minimum age of the subjects in this study was 11 years old, which does not cover the subject population in the study of Governder and Mars. Perhaps a study that addresses a wider age range would provide more accurate information.

The maximum ambient noise monitored in this study was 66.2 ± 7.2 dB, which did not exceed the MPANLs specified in the instructions, for transient occurrences of high ambient noise, where KUDUwave pauses the test, allowing good correlation to be obtained between the results of the automated test performed in a general clinic room and the manual test in an acoustically insulated room. It has been shown that insert earphones, when used in combination with earmuffs, optimize ambient noise attenuation to a level where the total noise attenuation can exceed that of a single-walled sound-insulated room (Seluakumaran and Shaharudin, 2021). Because ambient noise can affect test results not only through air conduction, higher ambient noise can also affect results through bone conduction. Therefore, it is recommended that testing in a non-soundproofed environment be performed in a quiet room.

In our previous study, we performed a comparison of manual and automated tests under sound-isolation conditions and found that the reliability at 250 Hz and 8,000 Hz was worse than at other frequencies (Liu et al., 2022), however, this phenomenon did not occur in the present group of subjects, which may be related to the use of different headphones. In the previous study, insert headphones were used for automated audiometry and circumaural earphones were used for manual audiometry; in the present study, KUDUwave audiometer were used for both manual-and automated pure-tone audiometry, which eliminates the calibration differences that were introduced by two different devices, and could easily interpret some changes in hearing thresholds.

Study limitations.

One of the limitations of this study is that bone conduction threshold tests were not conducted on the reliability of automated pure tone audiometry. The relationship between bone and air conduction is an important basis for determining the presence or absence of conductive hearing loss, and a subsequent study will be conducted to investigate the clinical application of bone conduction for automated audiometry.

The ambient noise levels were recorded manually by a sound level meter, and were also continuously monitored by KUDUwave, however, the data from KUDUwave was not available. It would be useful to compare whether both the noise monitoring methods provided similar levels.

Although the sample size estimate indicated that the 83 initial subjects for this study met the requirements. However, the inclusion of a larger sample of subjects would have improved the credibility of this study. The insufficiently large sample size is a limitation of this study.

## 5 Conclusion

In this study, subjects were grouped according to age and hearing level, respectively. Automated pure-tone audiometry was performed in the general consultation room, and manual pure-tone audiometry was performed in the acoustic isolation room using KUDUwave audiometer. There was a good correlation between the automated and manual audiometric thresholds. Subjects’ reaction times increased with age, and reaction time measurements provided a basis for a more accurate parameter setting of the automated tests. In the case of individual subjects with high variability of results, quality control of the automated test needs to be increased so that such subjects can be screened out and transferred to manual audiometry. In conclusion, automated air conduction pure-tone audiometry has great potential to play a greater role, especially in economically underdeveloped areas, or in mass hearing screening scenarios.

## Data availability statement

The raw data supporting the conclusions of this article will be made available by the authors, without undue reservation.

## Ethics statement

The studies involving humans were approved by The Medical Ethics Committee for Clinical Research of Beijing Tongren Hospital. The studies were conducted in accordance with the local legislation and institutional requirements. Written informed consent for participation in this study was provided by the participants' legal guardians/next of kin.

## Author contributions

HL: Writing – original draft, Writing – review & editing. XF: Writing – original draft, Writing – review & editing. ML: Writing – original draft. SW: Writing – review & editing.
