# Supplementary material for: Comparisons of air-conduction hearing thresholds between manual and automated methods in a commercial audiometer
Source: Front Neurosci. 2023 Dec 21;17:1292395. doi: 10.3389/fnins.2023.1292395 (PMC10771286; doi:10.3389/fnins.2023.1292395)
Supplement: Supplementary file 1 [file Table_1.DOCX]

Supplementary Material

**Comparisons of air-conduction hearing thresholds between manual and automated methods in a commercial audiometer**

**Hui Liu^1, 2^, Xinxing Fu^1, 2, 3, 4*,^ Mohan Li^1, 2^, Shuo Wang^1,2*^**

*Corresponding Author: Xinxing Fu, Email: xinxing.fu@research.uwa.edu.au

*Corresponding Author: Shuo Wang, Email: shannonwsh@aliyun.com

# 1. Supplementary Tables

eTable 1. Protocols for manual and automated audiometry

|  | Manual procedure | Automated procedure |
| --- | --- | --- |
| Stimulus | Pure tone | Pure tone |
| Presentation duration of the stimulus | 1s | 1s |
| Response from subjects | Pressing and releasing a signal switch | Pressing and releasing a signal switch |
| Determining the correct response | Experience of the tester | Response within 2s after delivering pure tone |
| Initial intensity for the first frequency of each ear | 30dB HL | 30dB HL |
| Initial intensity for the subsequent frequencies | 10dB above the threshold of the previous frequency | 30dB HL |

eTable 2. Post-hoc power analysis

| Effect size | Significant level | Sample size | Power |
| --- | --- | --- | --- |
| 1.88 | 0.05 | 83 | 1 |

The following script was run in R environment

# Install and load the pwr package

install.packages("pwr")

library(pwr)

# Input parameters

effect_size <- 1.88 # Example effect size

alpha <- 0.05 # Significance level

observed_p <- 0.05 # Example observed p-value

sample_size <- 83 # Example sample size

# Run post-hoc power analysis

power_analysis <- pwr.t.test(d = effect_size, n = sample_size, sig.level = alpha,

alternative = "two.sided", p = observed_p)

# Print power

cat("Power:", power_analysis$power, "\n")

eTable 3. Participants characteristics

| Characteristics | N | % |
| --- | --- | --- |
| Sex |  |  |
| Male | 41 | 49.4 |
| Female | 42 | 50.6 |
| Age (years) |  |  |
| 11-20 | 6 | 7.2 |
| 21-40 | 15 | 18.1 |
| 41-60 | 27 | 32.5 |
| 60-80 | 32 | 38.6 |
| >80 | 3 | 3.6 |

eTable 4. False positives of automated audiometry

|  | Rate of false positive (%) | | | | | | |
| --- | --- | --- | --- | --- | --- | --- | --- |
|  | 0 | ≤5 | ≤10 | ≤20 | ≤30 | ≤40 |  |
| Case (n) | 47 | 69 | 76 | 81 | 82 | 83 |  |
| Percentage (%) | 57 | 83 | 92 | 98 | 99 | 100 |  |

eTable 5. Response time of automated and manual pure-tone audiometry

|  | Age groups | | |  |
| --- | --- | --- | --- | --- |
|  | ＜40 years  (n=21) | 40～60 years  (n=27) | ＞60 years  (n=35) | Total  （n=83） |
| M Response time in ms (SD) | 791.5 (181.2)* | 900.4 (190.9) | 1063.1 (332.3)* | 941.5 (279.3) |

* Indicate significance between the group below 40 years and the group above 60 years (P＜0.01)

# 2. Supplementary Figures


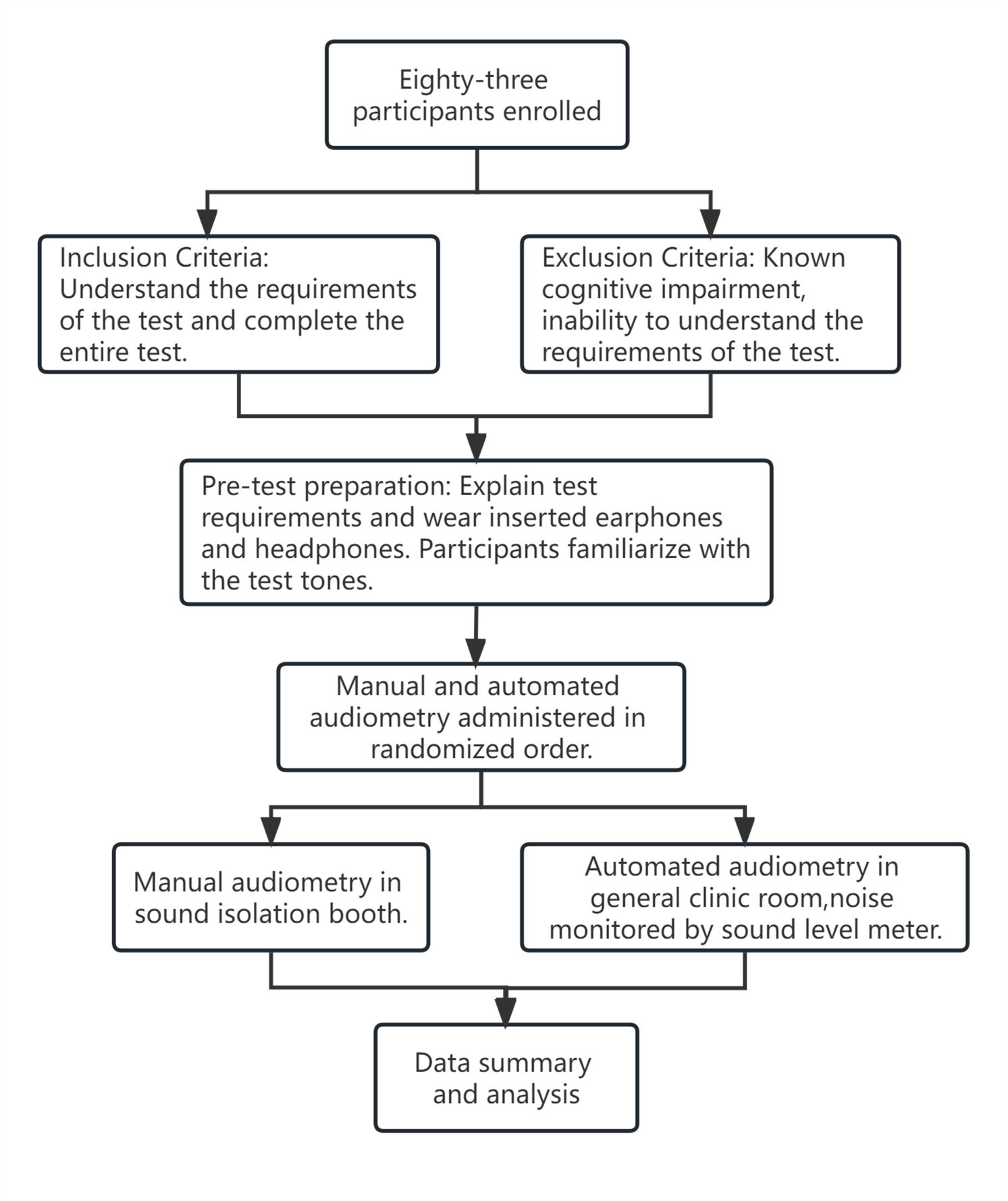


eFigure 1. Flowchart of test procedure


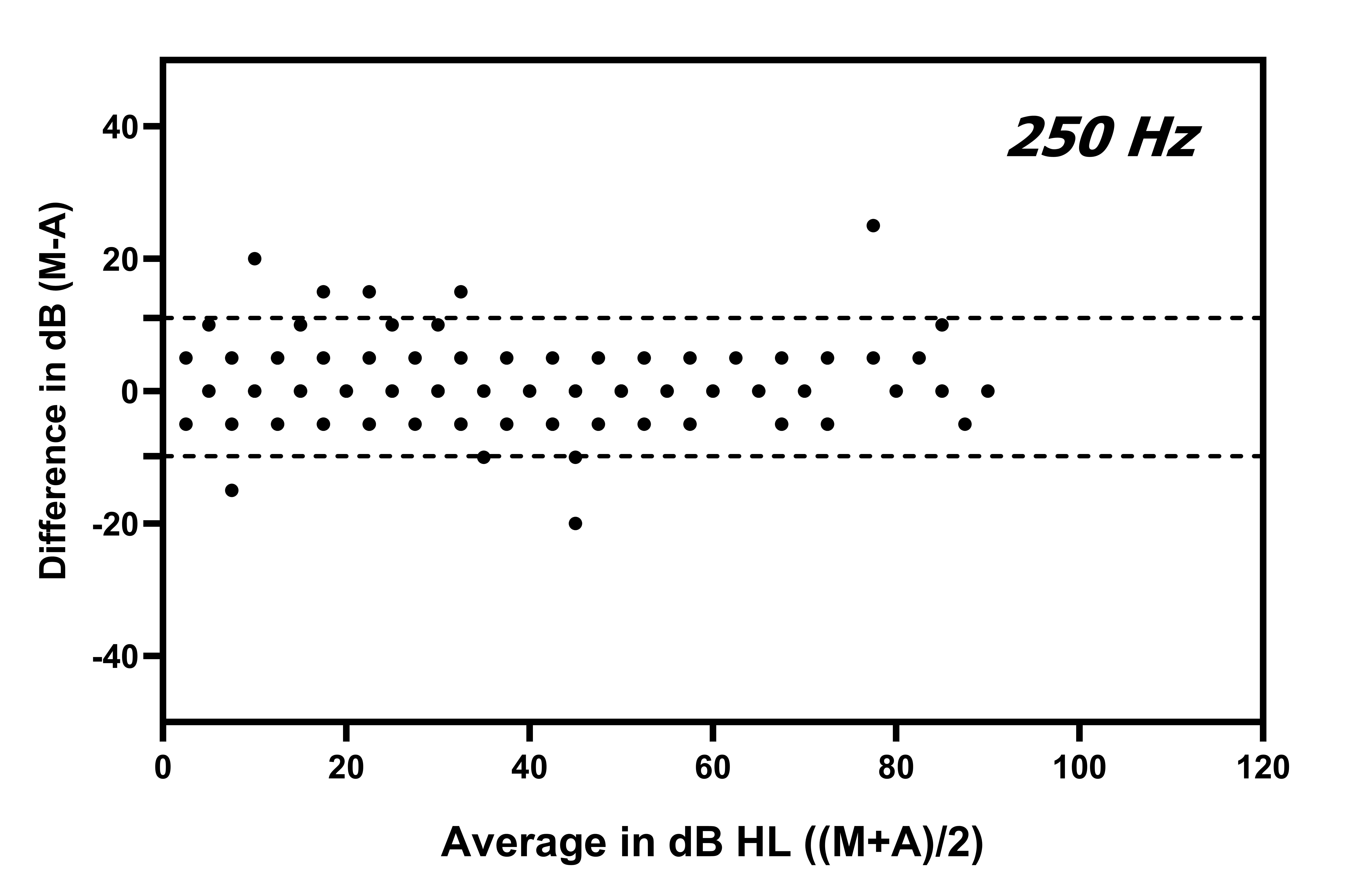


**eFigure 2-A.** Bland-Altman plots of the results of the automated and manual pure-tone audiometry at the frequency of 250 Hz.


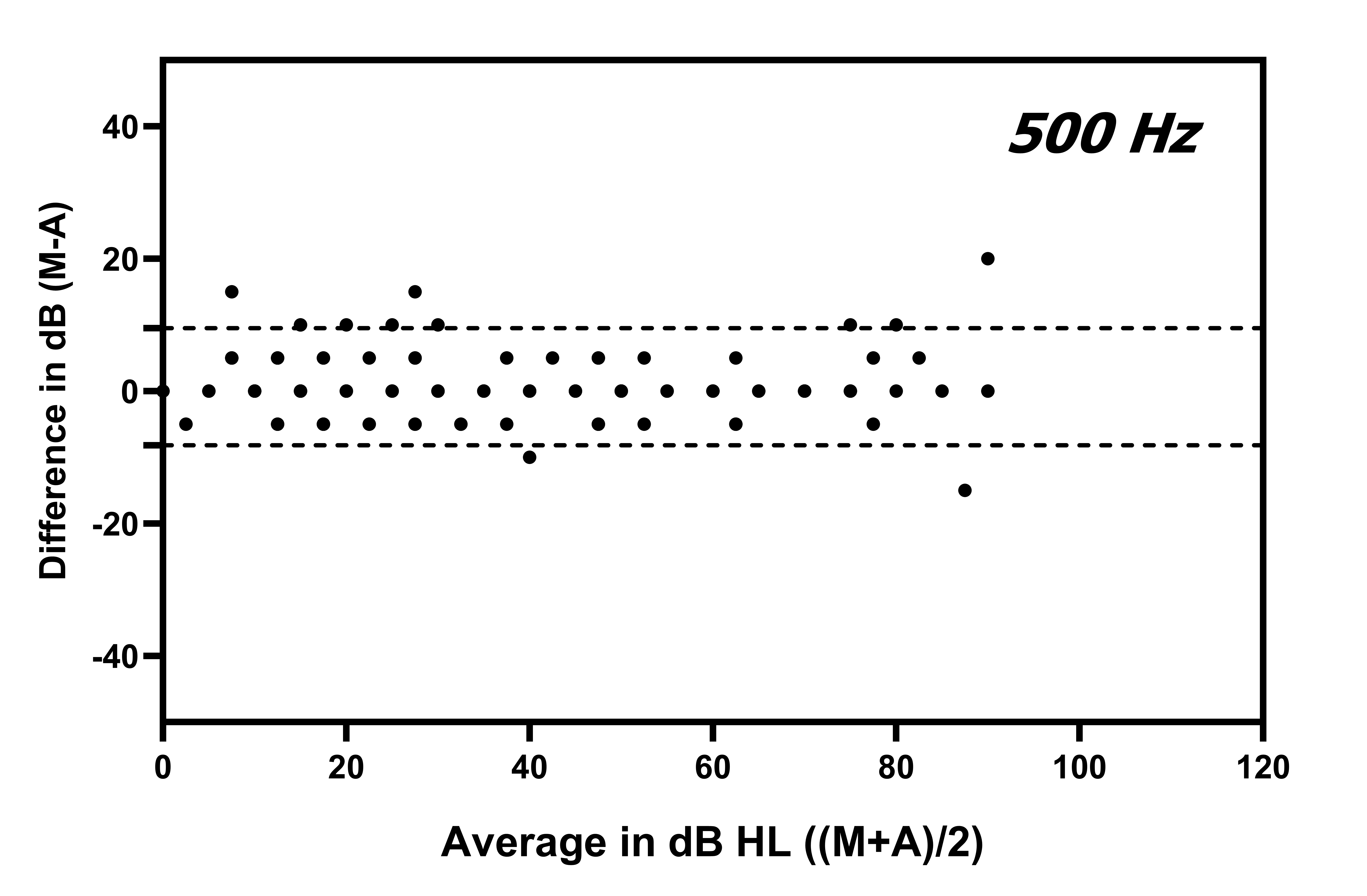


**eFigure 2-B.** Bland-Altman plots of the results of the automated and manual pure-tone audiometry at the frequency of 500 Hz.


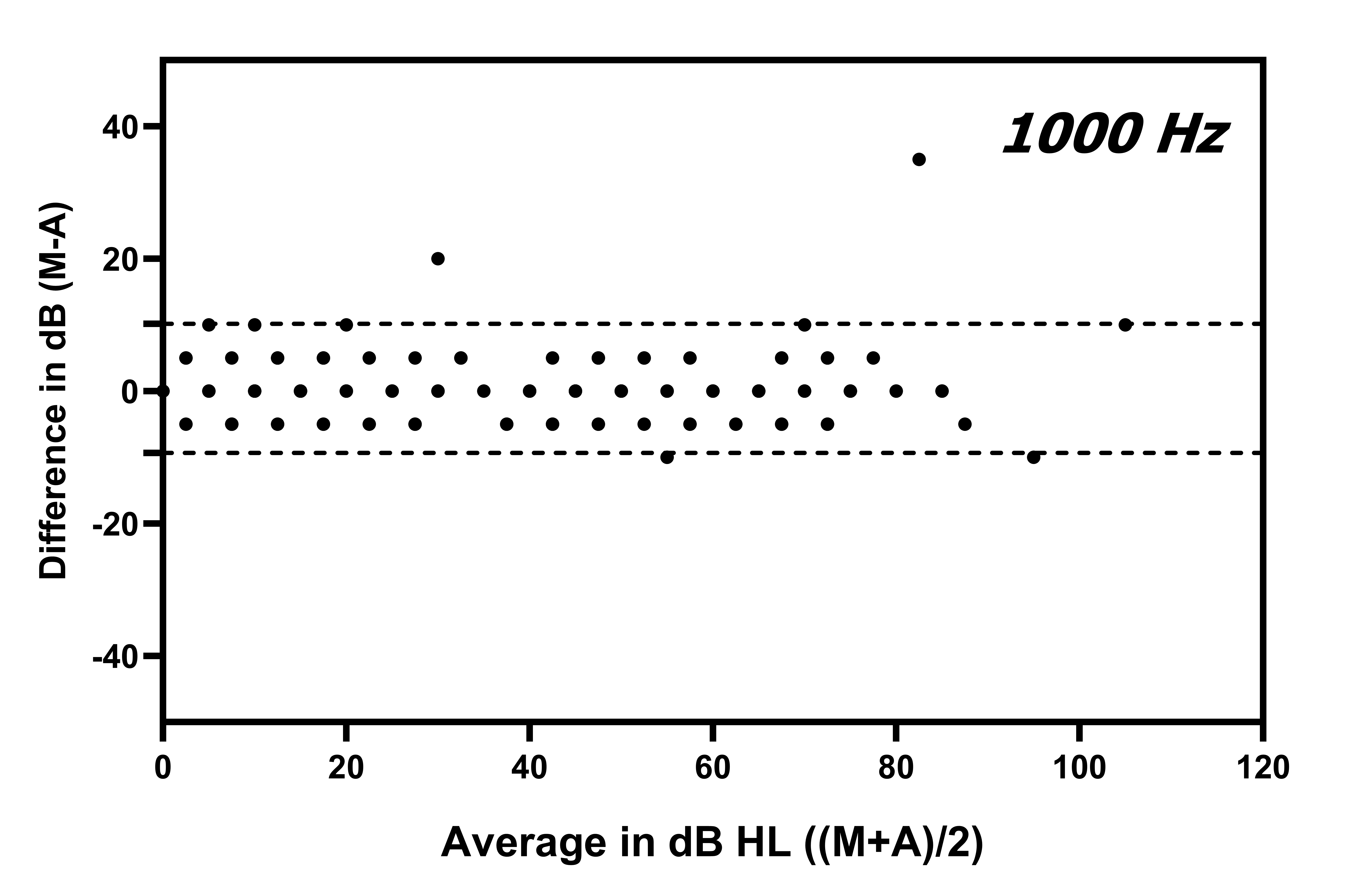


**eFigure 2-C.** Bland-Altman plots of the results of the automated and manual pure-tone audiometry at the frequency of 1000 Hz.


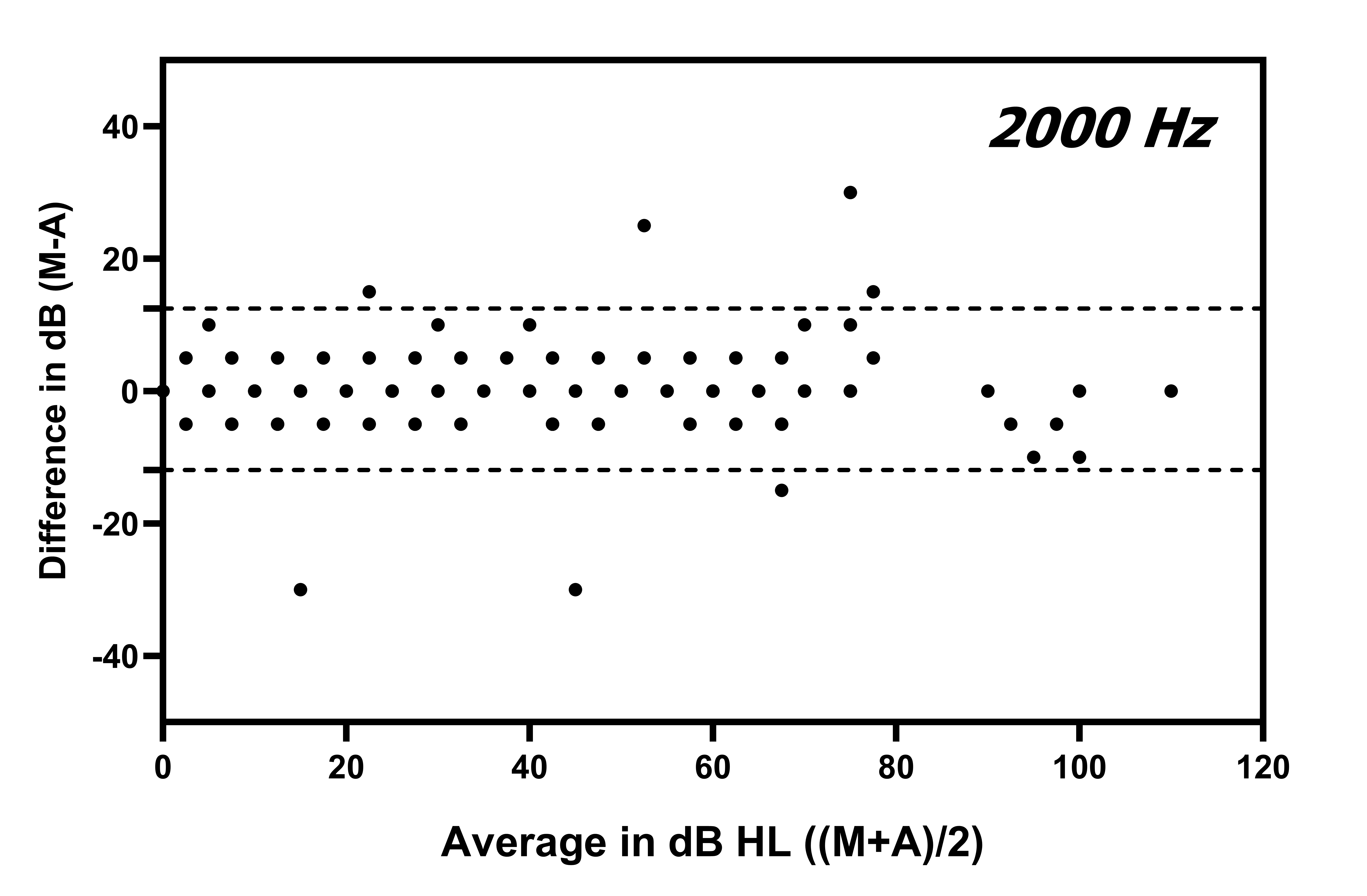


**eFigure 2-D.** Bland-Altman plots of the results of the automated and manual pure-tone audiometry at the frequency of 2000 Hz.


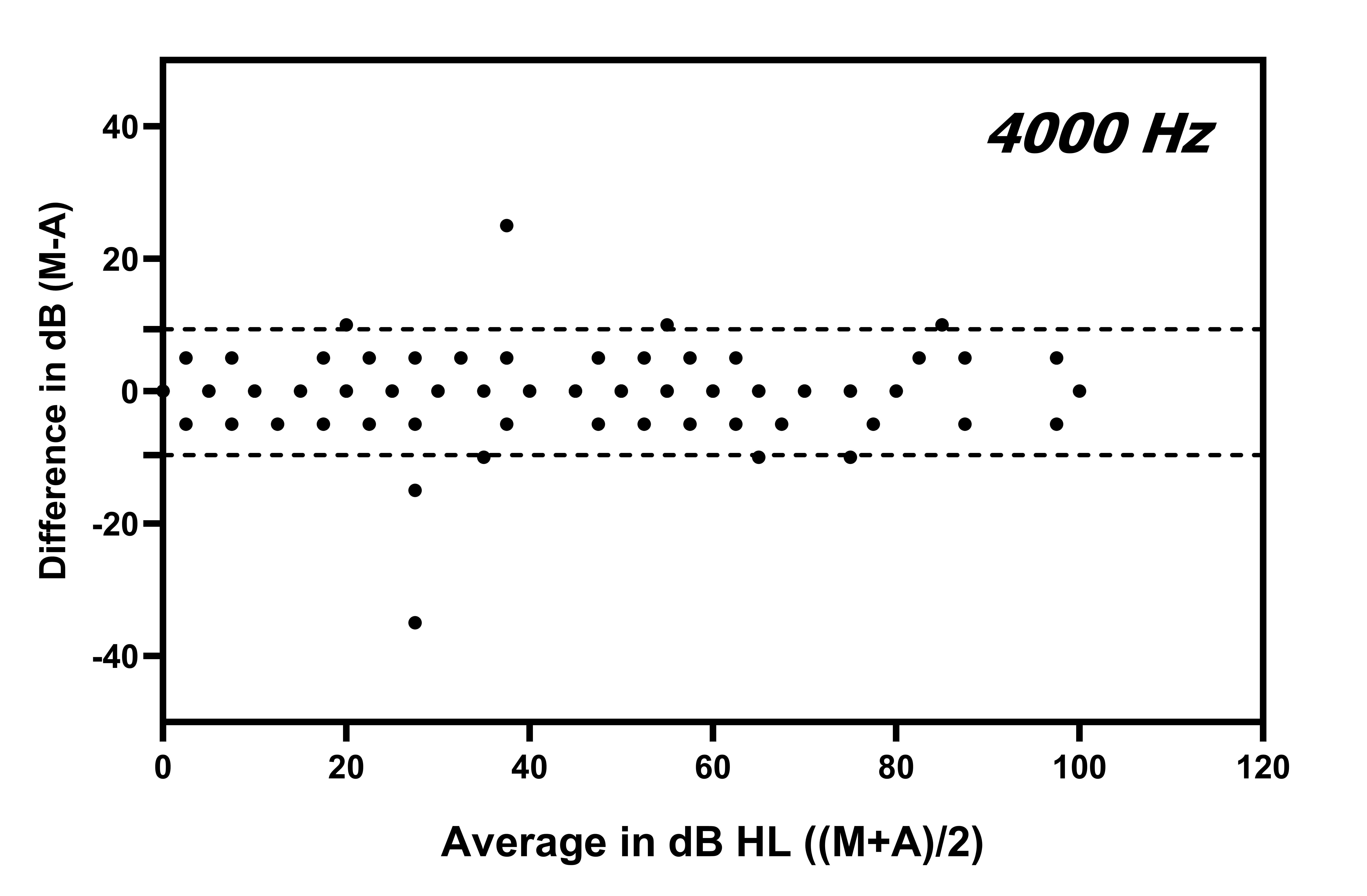


**eFigure 2-E.** Bland-Altman plots of the results of the automated and manual pure-tone audiometry at the frequency of 4000 Hz.

**
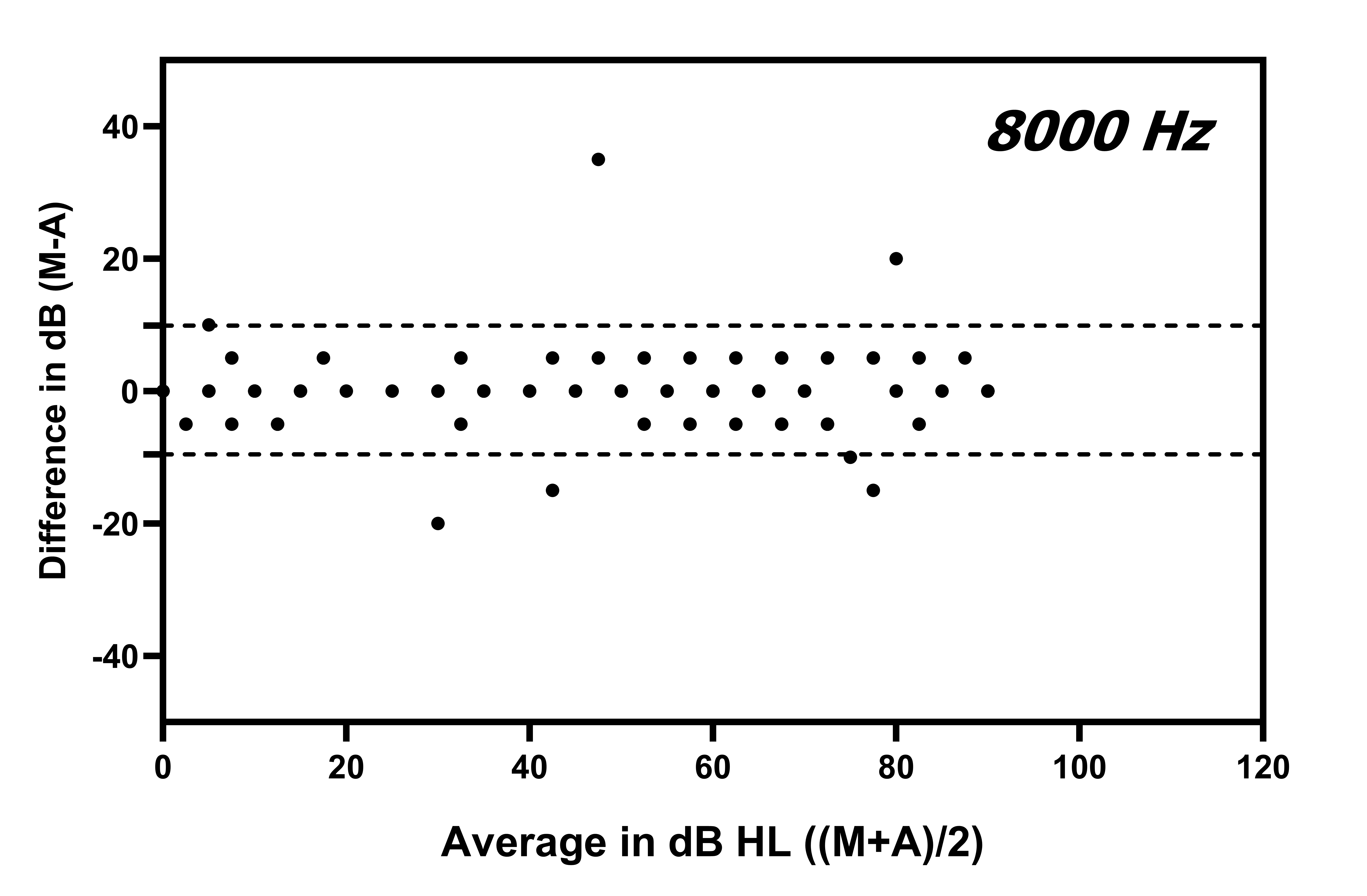
**

**Figure 2-F.** Bland-Altman plots of the results of the automated and manual pure-tone audiometry at the frequency of 8000 Hz.


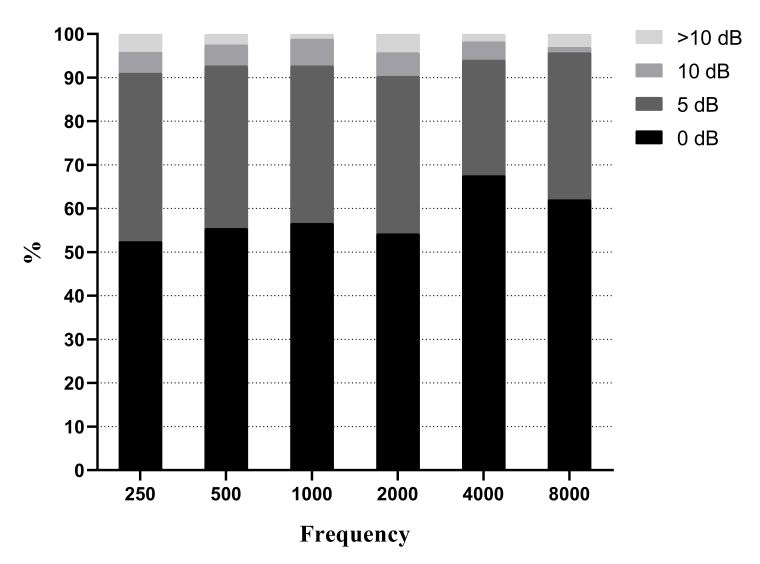


eFigure 3. Distribution of the absolute differences between automated and manual test thresholds at each frequency.
